# *RET*融合阳性晚期非小细胞肺癌治疗进展

**DOI:** 10.3779/j.issn.1009-3419.2021.101.43

**Published:** 2021-12-20

**Authors:** 青云 高, 俊威 苏, 法嫚 肖, 晓程 林, 衿记 杨

**Affiliations:** 510080 广州，广东省心血管病研究所，广东省人民医院，广东省医学科学院，广东省肺癌研究所，广东省肺癌转化医学重点实验室 Guangdong Cardiovascular Institute, Guangdong Provincial People's Hospital, Guangdong Academy of Medical Sciences, Guangdong Lung Cancer Institute, Guangdong Provincial Key Laboratory of Translational Medicine in Lung Cancer, Guangzhou 510080, China

**Keywords:** *RET*融合, 肺肿瘤, 靶向治疗, *RET* fusion, Lung neoplasms, Targeted therapy

## Abstract

转染重排（rearranged during transfection, *RET*）融合阳性发生于0.7%-2%的非小细胞肺癌（non-small cell lung cancer, NSCLC）。*RET*基因与其他结构域之间的融合代表了NSCLC独特的生物学和临床病理学亚型。近些年以来，*RET*融合阳性晚期NSCLC治疗领域取得了重要进展。传统化疗能够带来一定的临床获益。在靶向药物临床应用之前或不适用的情况下，以铂类为基础的系统性化疗方案是患者初始治疗选择。免疫治疗在*RET*融合阳性人群中的报告数据较少且通常结果较差，不推荐免疫单药或免疫联合化疗方案作为这类患者的系统性治疗。多靶点激酶抑制剂在这类人群中的重新应用能够取得一定的治疗反应，目前仅卡博替尼和凡德他尼推荐在有限条件下作为初始或后续治疗。然而，依然存在不能满足的临床需求。普拉替尼（Pralsetinib）和塞尔帕替尼（Selpercatinib）作为RET选择性酪氨酸激酶抑制剂（tyrosine kinase inhibitor, TKI），显著改善了*RET*融合阳性NSCLC患者生存及预后。普拉替尼和塞尔帕替尼已确立成为这类患者一线或后续治疗的首选方案。与其他TKI治疗时观察到的类似，RET靶向抑制治疗也会导致耐药问题，而获得性耐药的出现将影响治疗的远期有效性，并限制后续的药物选择。因此，本文将围绕RET融合阳性晚期NSCLC治疗进展进行综述。

## Abstract (en)

Rearranged during transfection (*RET*) fusions are found in 0.7% to 2% of non-small cell lung cancer (NSCLC). Fusions between *RET* gene and other domains represent the distinct biological and clinicopathological subtypes of NSCLC. Recent years have witnessed the remarkable advancement of *RET* fusion-positive advanced NSCLC therapy. Conventional chemotherapy produced moderate clinical benefits. Prior to the introduction of targeted therapy or in the context of unavailability, platinum-based systemic regimens are initial therapy options. Immunotherapy predicted minimal response in the presence of *RET* fusions while currently available data have been scarce, and the single-agent immunotherapy or in combination with chemotherapy regimens are not recommended as initial systemic therapy in this population. The repurpose of multi-target kinase inhibitors in patients with *RET* fusion-positive NSCLC showed encouraging therapeutic activity, with only cabozantinib and vandetanib being recommended as initial or subsequent options under certain circumstances. However, there are still unmet clinical needs. Pralsetinib and selpercatinib have been developed as tyrosine kinase inhibitors (TKI) selectively targeting RET variation of fusions or mutations, and both agents significantly improved the prognosis of patients with *RET* fusion-positive NSCLC. Pralsetinib and selpercatinib have been established as preferred first-line therapy or subsequent therapy options. As observed with other TKIs treatment, resistance has also been associated with RET targeted inhibition, and the acquired resistance eventually affect the long-term therapeutic effectiveness, leading to limited subsequent treatment options. Therefore, it is essential to identify resistance mechanisms to TKI in *RET* fusion-positive advanced NSCLC to help reveal and establish new strategies to overcome resistance. Here, we review the advances in the treatment of *RET* fusion-positive advanced NSCLC.

## 1 引言

转染重排（rearranged during transfection, *RET*）原癌基因编码一种受体酪氨酸激酶，对胚胎发生时期肠神经系统和肾脏的发育至关重要^[1]^，但在正常肺组织的表达水平普遍很低^[2]^。*RET*基因位于染色体10q11.2，其表达由转录因子Sp家族（Sp1, Sp3）的DNA结合蛋白或早期生长反应蛋白1（early growth response 1, EGR1）等介导^[3]^。RET编码跨膜受体酪氨酸激酶（receptor tyrosine kinase, RTK），独特结构包含钙粘蛋白样结构域、富半胱氨酸结构域、跨膜结构域和酪氨酸激酶结构域^[4]^。RET受体酪氨酸激酶参与细胞生存、增殖、分化、迁移以及趋化等^[5]^。

*RET*作为原癌基因而被发现，是一种癌症驱动基因。*RET*基因融合或点突变可引起非配体依赖性激活，嵌合融合蛋白的形成为其临床表现形式之一^[6]^。*RET*与多种伴侣基因发生融合后，持续激活细胞外信号调节激酶（extracellular signal-regulated kinase, ERK）和磷脂酰肌醇3-激酶（phosphoinositide 3-kinase, PI3K）-蛋白激酶B（protein kinase B, AKT）等信号级联反应^[7]^，进而促进胞内酪氨酸激酶结构域的自我磷酸化、信号传导增强以及癌变转化^[8, 9]^。首例检测出因染色体倒位而形成RET基因融合的肺癌病例报告于2011年，利用全基因组测序在1例33岁非吸烟肺腺癌韩国男性患者肝脏转移灶中发现驱动蛋白家族成员5B（*KIF5B*）-*RET*^[10]^。

*RET*融合发生于0.7%-2%的非小细胞肺癌（non-small cell lung cancer, NSCLC），在腺癌组织学类型中检出率更高^[11-15]^。常见的融合伴侣包括*KIF5B*（约70%）、卷曲螺旋结构域蛋白6（coiled-coil domain-containing protein 6, *CCDC6*）（约20%），检出率低于2%的包括核受体共激活因子4（nuclear receptor coativator 4, NCOA4）、红细胞生成素肝细胞受体A（erythropoietin-producing hepatocyte receptor A, EPHA）以及磷脂酰肌醇结合网格蛋白组装蛋白（recombinant phosphatidylinositol binding clathrin assembly protein, PICALM）^[16]^。含三联基元33（tripartite motif-containing, TRIM33）也有报告^[17]^。

*RET*基因与其他基因结构域之间会发生融合，由此产生了NSCLC独特的生物学和临床病理学亚型。与非*RET*融合阳性NSCLC相比，这类患者往往年龄较轻（平均年龄：62.9岁*vs* 67.2岁）、从不吸烟（非吸烟者比例：63.0% *vs* 18.1%）、以腺癌居多（非鳞癌比例：100% *vs* 79.4%）、体力状态较好^[18]^，常伴脑转移（Ⅳ期患者确诊时的脑转移发生率为25%，终生患病率为46%）^[19]^。中国患者特征与西方人群类似，确诊时脑转移比例在17.7%-27.8%^[20, 21]^。

根据最《美国国家综合癌症网络（National Comprehensive Cancer Network, NCCN）肿瘤临床管理实践指南：非小细胞肺癌》2021年第5版，*RET*可通过荧光原位杂交（fluorescence *in situ* hybridization, FISH）分离探针或实时逆转录-聚合酶链反应（reverse transcription-polymerase chain reaction, RT-PCR）检测^[22]^。然而，某些非经典融合类型尤其涉及罕见融合伴侣时可能无法分辨及检出。基于下一代测序（next generation sequencing, NGS）的方法特异性高，以RNA为基础的NGS在识别融合伴侣方面优于以DNA为基础的NGS^[22]^。2021年6月，《中国非小细胞肺癌*RET*基因融合临床检测专家共识》发表，强烈推荐所有病理诊断为腺癌的晚期NSCLC患者进行*RET*基因检测，同时推荐经活检组织病理学证实为非腺癌的晚期NSCLC、对表皮生长因子受体（epidermal growth factor receptor, EGFR）-酪氨酸激酶抑制剂（tyrosine kinase inhibitor, TKI）、间变性淋巴瘤激酶（anaplastic lymphoma kinase, ALK）-TKI耐药的患者、术后明确为浸润性腺癌的患者进行*RET*基因检测^[23]^。该指南推荐根据送检标本的类型（如肿瘤组织、细胞学、液体）、数量与质量、所检基因变异类型和数量以及实验室条件等合理选择检测方式，NGS-DNA/RNA以及RT-PCR均为强烈推荐的检测方法，必要时行多平台检测互补和验证^[23]^。在2021年9月发布的中国临床肿瘤学会（Chinese Society of Clinical Oncology）《非小细胞肺癌肺癌诊疗指南（2021版）》中，对于不可手术Ⅲ期及Ⅳ期NSCLC，进一步将*RET*融合基因检测从Ⅱ级推荐提升为Ⅰ级推荐，推荐与*EGFR*、*ALK*、*ROS1*肺癌驱动基因同时检测^[24]^。

就*RET*融合阳性晚期NSCLC治疗策略而言，2021年第5版NCCN NSCLC指南推荐一线/后线治疗药物包括普拉替尼、塞尔帕替尼、卡博替尼、凡德他尼等，其中前两者代表了*RET*融合阳性肿瘤靶向治疗领域的崭新突破，推荐作为首选的一线用药方案^[22]^。2021版CSCO NSCLC诊疗指南推荐使用普拉替尼、塞尔帕替尼治疗*RET*融合基因阳性Ⅳ期NSCLC^[24]^。靶向药物治疗进展后，含铂化疗方案或免疫治疗可作为后线选择可作为后线选择^[22, 24]^。本文将主要针对RET融合阳性晚期NSCLC人群的药物治疗进展进行回顾。

## 2 *RET*融合阳性晚期NSCLC药物治疗进展

### 2.1 靶向治疗

#### 2.1.1 多靶点激酶抑制剂（multikinase inhibitor, MKI）

RET受体酪氨酸激酶与其他酪氨酸激酶在激酶域结构上有相似之处^[25]^。早期针对*RET*融合阳性的肿瘤靶向治疗研究主要集中在具有非选择性RET抑制活性的MKI上，卡博替尼和凡德他尼为其中的代表，既抑制*RET*融合也抑制VEGFR/EGFR等其他激酶。

2017年，一项全球多中心的RET注册研究（GLORY）^[16]^回顾性收集了RET融合阳性晚期NSCLC使用多种MKI的疗效。对于既往未接受过RET-TKI治疗的患者，卡博替尼组（*n*=19）获得的ORR为37%，凡德他尼组（*n*=11）为18%，舒尼替尼组（*n*=9）为22%，尼达尼布组（*n*=2）报告了1例完全缓解，索拉非尼组（*n*=2）、阿来替尼组（*n*=2）、仑伐替尼组（*n*=2）、帕纳替尼组（*n*=2）、瑞戈非尼组（*n*=1）均未观察到缓解^[16]^。截至分析时，仅15%的患者仍在继续治疗，85%停止治疗^[16]^。

针对26例转移性或不可切除的*RET*融合阳性肺腺癌患者的Ⅱ期研究中，卡博替尼获得的总缓解率（overall response rate, ORR）为28%。73%的患者因药物相关不良事件（adverse event, AE）需要减量。最常见的3级药物相关AE为无症状的脂肪酶升高（15%）^[17]^。

一项Ⅱ期研究^[26]^探索了凡德他尼在转移性或复发的RET融合阳性NSCLC韩国患者中的疗效。既往接受过多次化疗且可评估的17例患者中，ORR为18%，疾病控制率（disease control rate, DCR）65%。无疾病进展生存期（progression free survival, PFS）4.5个月，总生存期（overall survival, OS）11.6个月。凡德他尼在19例经治晚期*RET*融合阳性NSCLC日本患者中显示了更好的疗效，ORR为53%，PFS为4.7个月，OS为11.1个月^[27]^。大多数药物相关AE为轻度。3级或4级AE包括高血压等^[26, 27]^。

鉴于MKI有限的疗效以及较高的不良反应发生率（表 1）^[16, 17, 26-30]^，目前仅卡博替尼和凡德他尼获得NCCN指南推荐在有限条件下治疗转移性RET融合阳性NSCLC^[22]^。然而，二者在原有适应证基础上重新用于*RET*融合阳性NSCLC的探索并未达到理想目标，出现治疗应答的病例均为部分缓解，且伴随的VEGFR2抑制带来的靶外效应影响了用药疗程的完成。另一方面，上述早期研究结果证实了RET可作为临床上行之有效的靶点，促进了从MKI到特异性RET-TKI的转换。

**表 1:** MKIs治疗*RET*融合阳性NSCLC研究 Clinical trials of multi-kinase inhibitors in the treatment of *RET* fusion-positive NSCLC

Multi-kinase inhibitors	Target	Efficacy	Adverse effects	Reference
Cabozantinib	VEGFR, C-Met, RET	ORR: 28%-37%, PFS: 3.6 mon-5.5 mon, OS: 4.9 mon-9.9 mon	73% of pts had dose reductions due to intolerable G2-3 TRAEs, 8% discontinued due to TRAEs	Drilon A, 2016^[17]^ Gautschi J, 2017^[16]^
Vandetanib	VEGFR 2, VEGFR 3, EGFR, RET	ORR: 18%-53%, PFS: 2.9 mon-4.7 mon, OS: 10.2 mon-11.6 mon	22% to 53% of pts had dose reductions due to TRAEs, 21% discontinued due to TRAEs, 11% experienced serious TRAEs	Lee SH, 2017^[25]^ Yoh K, 2017^[26]^ Gautschi O, 2017^[16]^
Sunitinib	c-KIT, VEGFR 1-3, PDGFRβ, FLT3, RET	ORR: 22%, PFS: 2.2 mon, OS: 6.8 mon	NA	Gautschi O, 2017^[16]^
Sorafenib	CRAF, BRAF, KIT, FLT3, VEGFR, RET	ORR: 0%, PFS: NA, OS: NA	NA	Horiike A, 2016^[27]^ Gautschi O, 2017^[16]^
Alectinib	ALK, RET, FLT3, kinase 2, leukocyte receptor tyrosine kinase	ORR: 0%-33%, PFS: NA, OS: NA	NA	Lin JJ, 2016^[28]^ Gautschi O, 2017^[16]^
Lenvatinib	VEGFR 1-3, FGFR 1-4, PDGFRα, c-KIT, RET	ORR: 0%-16%, PFS: NA, OS: NA	64% of patients had dose reductions due to TRAEs, 20% discontinued due to TRAEs, 12% experienced fatal AEs	Velcheti V, 2016^[29]^ Gautschi O, 2017^[16]^
NSCLC: non-small cell lung cancer; AE: adverse events; EGFR: epidermal growth factor receptor; FGFR: fibroblast growth factor receptor; G: grade; mon: month; MKI: multi-kinase inhibitor; NA: not available; ORR: objective response rate; OS: overall survival; PDGFR: platelet-derived growth factor receptor; PFS: progression-free survival; RET: rearranged during transfection; TRAE: treatment-related AE; VEGFR: vascular endothelial growth factor receptor.				

#### 2.1.2 RET-TKI

普拉替尼（BLU-667）和塞尔帕替尼（LOXO-292）为口服、强效、高度选择性RET-TKI，治疗*RET*融合阳性晚期NSCLC均获得了优异的疗效。普拉替尼为新型小分子激酶抑制剂，对野生型*RET*、致癌性*RET*融合（包括最常见的*KIF5B-RET*和*CCDC6-RET*）以及突变（*RET* V804L、*RET* V804M和*RET* M918T）均有高度效力和选择性，半数抑制浓度（50% concentration of inhibition, IC_50_）均 < 0.5 nmol/L^[31, 32]^。普拉替尼为携带*RET*融合的NSCLC患者带来持久的缓解且无显著的靶外毒性反应^[33]^。另一高度选择性ATP竞争性RET-TKI为塞尔帕替尼，该药对包括获得性耐药突变在内的多种*RET*变异具有纳摩尔级的效力^[31]^。塞尔帕替尼也具有药代学优势，如高生物利用率（平均绝对值为73%）、对中枢神经系统的显著渗透力以及较少的药物相互作用^[34]^。二者均可以克服MKI治疗时发生的V804M等常见门卫突变。

全球多中心的单臂Ⅰ期/Ⅱ期ARROW临床研究评估了普拉替尼治疗包括NSCLC在内的*RET*变异实体瘤的疗效和安全性^[35]^。2021年美国临床肿瘤学会（American Society of Clinical Oncology, ASCO）年会报道的更新数据显示，对于可评估的RET融合阳性晚期NSCLC，随访17.1个月时，普拉替尼初始治疗获得的ORR为79%，完全缓解（complete response, CR）率为6%，中位缓解持续时间（duration of response, DoR）尚未达到（*n*=68）；中位PFS为13.0个月（*n*=75）。普拉替尼治疗铂类化疗经治患者ORR为62%，CR率为4%，DoR为22.3个月（*n*=126）；中位PFS为16.5个月（*n*=136）^[35]^。9例患者基线期伴可测量脑转移灶，颅内病灶ORR为56%，其中3例达到颅内CR（33%）^[36]^。普拉替尼400 mg *qd*治疗*RET*融合阳性晚期NSCLC耐受性良好，大部分治疗相关AE为1级-2级，普拉替尼治疗相关的最常见≥3级AE包括中性粒细胞减少症（20%）、贫血（13%）、高血压（12%）等^[35]^。基于ARROW研究结果，普拉替尼于2020年9月获得了美国食品药品监督管理局（U.S. Food and Drug Administration, FDA）批准作为*RET*融合阳性晚期NSCLC一线或后线治疗，并于2021年3月获得中国国家药品监督管理局附条件批准，用于既往接受过含铂化疗的*RET*基因融合阳性的局部晚期或转移性NSCLC成人患者的治疗。

全球性Ⅰ期/Ⅱ期临床研究LIBRETTO-001评估了塞尔帕替尼治疗晚期*RET*异常实体瘤患者的疗效和安全性^[37, 38]^。数据显示，塞尔帕替尼初始治疗*RET*融合阳性NSCLC（*n*=48）中位随访9.8个月时ORR为85%，CR率为2%；DoR尚未达到（中位随访15.7个月）；中位PFS尚未达到，1年PFS率为68%（中位随访10.8个月）；2年OS率为88%（中位随访12.6个月）^[38]^。塞尔帕替尼治疗铂类经治患者（*n*=105）中位随访15.7个月时ORR为64%，CR率为3%；DoR为17.5个月（中位随访15.7个月）；中位PFS为19.3个月，1年PFS率为66%（中位随访16.8个月）；2年OS率为68%（中位随访19.9个月）^[38]^。基线伴可测量脑转移灶的患者中（*n*=11），颅内ORR为91%（10/11）^[37]^。塞尔帕替尼治疗相关的最常见≥3级AE包括高血压（12%）、丙氨酸氨基转移酶（alanine transaminase, ALT）升高（8%）、天冬氨酸转氨酶（aspartate aminotransferase, AST）升高（7%）等^[38]^。基于LIBRETTO-001报告结果，塞尔帕替尼于2020年5月被美国FDA批准一线或后线治疗RET融合阳性晚期NSCLC。

以上研究在中国亚组中得到类似的结果。根据2021年9月初世界肺癌大会（World Conference on Lung Cancer, WCLC）最新报告，ARROW研究中，中位随访8.2个月时，普拉替尼初始治疗中国患者获得的ORR为80%，CR率为6.7%（*n*=30）；中位随访17.0个月时，普拉替尼治疗铂类化疗经治中国患者的ORR为66.7%，CR率为3.0%（*n*=33）；最常见的3级-4级治疗相关AE包括中性粒细胞计数减少（33.8%）、贫血（32.4%）以及血肌酐磷酸激酶升高（17.6%）^[39]^。同样地，2021年WCLC公布的LIBRETTO-001中国亚组数据^[40]^显示，中位随访10.3个月时，塞尔帕替尼初始治疗（*n*=11）获得的ORR为90.9%，CR率为18.2%；铂类/免疫制剂/MKI经治患者（*n*=36）的ORR为58.3%，CR率为2.8%；最常见的≥3级治疗相关AE包括ALT升高（15.6%）、AST升高（15.6%）以及高血压（15.6%）。

普拉替尼与塞尔帕替尼治疗*RET*融合NSCLC均具有强效且持续的缓解，兼有良好的颅内活性。NCCN NSCLC指南（2021年第5版）推荐普拉替尼和塞尔帕替尼作为*RET*融合阳性转移性NSCLC一线或后续治疗首选；若一线治疗期间发现存在RET融合，可继续完成原系统治疗或中断治疗接受普拉替尼与塞尔帕替尼治疗^[22]^。

### 2.2 化疗

有研究^[20]^提示，*RET*融合阳性NSCLC对以培美曲塞为基础的化疗方案敏感。一项多中心、回顾性分析显示，纳入62例*RET*融合阳性晚期NSCLC，接受培美曲塞为基础的化疗（联合铂类或单药），ORR和DCR分别为50%、90.9%；其他化疗方案的ORR和DCR分别为44.4%、77.8%；以培美曲塞为基础的化疗显著延长PFS（9.2个月*vs* 5.2个月，*P*=0.007），并有延长OS的趋势（35.2个月*vs* 22.6个月，*P*=0.052）。

GLORY全球研究^[16]^中，108例*RET*融合阳性晚期NSCLC患者接受一线化疗，其中84例为铂类两联，66例为铂类+培美曲塞，获得的最佳缓解分别为52%、51%、49%，中位PFS分别为6.6个月、7.8个月、6.4个月，中位OS分别为23.6个月、24.8个月、23.6个月。

整体而言，化疗能够带来一定的临床获益。在靶向药物临床可及前或不适用的情况下，以铂类为基础的方案是患者有限的治疗选择之一。

### 2.3 免疫治疗

免疫检查点抑制剂（immune checkpoint inhibitor, ICI）已成为驱动基因阴性转移性NSCLC的标准治疗^[22]^。但免疫治疗对*EGFR*、*ALK*等驱动基因突变阳性患者的疗效不佳。免疫治疗*RET*融合阳性NSCLC的数据有限，样本量小。大多数*RET*融合阳性NSCLC程序性死亡配体1（programmed death-ligand 1, PD-L1）表达水平低，肿瘤突变负荷低^[41]^。

针对晚期RET融合阳性肺癌的回顾性研究^[40]^显示，在26例有足够组织用于PD-L1检测的患者中，肿瘤PD-L1表达0%、1%-49%的比例分别为58%、23%；在44例有足够组织用于肿瘤突变负荷分析的患者中，中位肿瘤突变负荷为1.76突变/Mb，低于*RET*野生型的中位值5.27突变/Mb。16例患者接受了免疫治疗，中位线数为2^[41]^。无论抗程序性死亡分子1（programmed death-1, PD-1）/程序性死亡受体-配体1（programmed cell death-ligand 1, PD-L1）单克隆抗体（单抗）单用还是联合抗细胞毒性T淋巴细胞相关抗原4（cytotoxic T lymphocyte-associated antigen-4, CTLA4）单抗治疗，均未观察到临床和/或病理学缓解病例。患者PFS为3.4个月^[41]^。

IMMUNOTARGET注册研究分析了来自10个国家的接受ICI单药治疗的晚期NSCLC人群，其中*RET*亚组（*n*=16）ORR仅为6%，PFS为2.1个月^[42]^。

GFPC 01-2018回顾性研究^[43]^报告了免疫治疗在真实世界RET融合NSCLC队列中高达37.5%的ORR和7.6个月的PFS，其中56%的纳入分析的患者（*n*=9）为PD-L1阴性，22%为PD-L1≥50%，且全部ICI用药为≥二线。

59例晚期或复发*RET*融合阳性NSCLC的韩国真实世界研究^[44]^中，凡德他尼组ORR为15.8%；以培美曲塞为基础的化疗（联合铂类或单用）患者ORR为63%，PFS为9.0个月，OS为24.1个月；免疫治疗组ORR为7.7%，PFS为2.1个月，OS为12.4个月。相反，针对45例中国患者的回顾性分析^[45]^表明，MKI *vs*化疗*vs* ICI治疗获得的PFS并无显著差异（3.8个月*vs* 3.5个月*vs* 2.5个月），接受ICI治疗的10例可评估患者ORR为20%。类似地，Hegde等^[46]^针对转诊至MD Anderson癌症中心参与Ⅰ期临床研究项目的70例*RET*融合阳性恶性肿瘤的回顾性分析发现，在29例NSCLC亚组，接受非ICI治疗的患者（*n*=13）中位至治疗终止时间长于ICI治疗，但差异无统计学意义（9.3个月*vs* 3.4个月，*P*=0.16）。该研究者认为这可能归因于非ICI治疗主要为效力不够强的老药MKI，而不是选择性RET抑制剂这类新药^[46]^。多变量分析中，非甲状腺髓样癌诊断为治疗终止风险升高的独立预测因素，非ICI治疗为治疗终止风险下降的独立预测因素^[46]^。

尽管ICI在肺癌治疗方面树立了新的里程碑，但基于目前有限的证据，免疫治疗*RET*融合阳性NSCLC患者的有效性不够理想，预测缺乏获益。NCCN指南指出，对于晚期或转移性NSCLC，若检出致癌基因（如*RET*融合阳性等），应禁忌PD-1/PD-L1抑制剂治疗，不推荐免疫单药或免疫联合化疗方案作为系统性初始治疗^[22]^。

## 3 探索与展望

目前*RET*融合阳性NSCLC治疗存在仍未满足的需求以及亟待解决的问题。随着强效RET-TKI的应用，获得性耐药的发生不可避免。这推动了相关耐药机制的探索以及应对策略的研究。

自发现以来，*RET*融合基因通常被认为与其他分子变异是互斥的^[47]^。但陆续有报告指出，少数病例中*RET*融合可能与*EGFR*^[11, 48, 49]^、*KRAS*^[48]^、*TP53*^[11]^、*AKT1*^[49]^、*MAP2K1*^[49]^、*CTNNB1*^[49]^突变重叠。另外，一些接受EGFR TKI治疗疾病进展的*EGFR*突变NSCLC患者中报告了*RET*融合^[50]^，提示*RET*融合可能为对EGFR抑制剂原发性或获得性耐药的潜在机制。对于*RET*融合阳性NSCLC中多种突变共存的现象，目前尚无确切结论。

### 3.1 靶向治疗耐药

RET抑制剂的耐药机制包括RET靶内和RET靶外旁路激活^[51]^。前者为靶点激酶内继发性（或获得性）耐药突变，在激酶抑制剂选择压力下动态进化，使激酶在用药情况下持续激活。后者指重新激活不同的细胞内通路，绕过靶向受体激酶介导的信号。

MKI耐药涵盖了上述全部机制，但以二次耐药变异的V804残基门卫突变为主（最为人们所熟知的为*RET* V804L/M^[52, 53]^），次之为错义突变S904F，其他如G810残基溶剂前沿突变、I788N体细胞突变目前多报告于临床前模型。就非RET变异而言，*MDM2*扩增被发现可导致*RET*重排阳性肺癌内在性与获得性耐药^[54]^，而其他旁路信号激活（如MAPK、EGFR、AXL）也仅见于临床前模型^[51]^。

鉴于不同于MKI与RET的结合模式，选择性RET-TKI能够避免门卫突变的干扰，但仍可发生非门卫继发性突变^[55]^，目前认为选择性RET-TKI耐药主要由独立于*RET*的靶外机制驱动，即以RET旁路激活为主，还包括RET位点的溶剂前沿突变。目前关于相关耐药机制的报道有限，对普拉替尼和塞尔帕替尼治疗耐药的20例*RET*融合阳性NSCLC肿瘤和血浆活检样本进行分析，检出3例（15%）*MET*扩增，2例（10%）溶剂前沿突变G810C/S耐药，1例（5%）*KRAS*扩增^[56]^。此前Solomon等^[57]^首次报告了利用ctDNA分析塞尔帕替尼治疗后疾病进展患者中发现G810溶剂前沿突变。另外，普拉替尼和塞尔帕替尼耐药细胞系中也发现了RET激酶域内铰链区（Y806C）和β2链区（V738A）突变^[55]^。研究证实，普拉替尼^[58, 59]^和塞尔帕替尼^[52, 60]^可以克服携带门卫突变的相关耐药。

### 3.2 联合治疗策略

#### 3.2.1 抗RET治疗+其他靶向制剂

2015年*RET*融合阳性NSCLC病例报告首次提供了凡德他尼+mTOR抑制剂依维莫司联合方案能够增强血脑屏障穿透的证据^[55]^，这在后来的研究中得到证实。凡德他尼+依维莫司为全部6例*RET*融合阳性NSCLC中均带来缓解，且对伴脑转移或卡博替尼难治性病例也表现出抗肿瘤活性^[61]^。前述LIBRETTO-001研究中有4例患者检出*MET*扩增，塞尔帕替尼+MET/ALK/ROS1抑制剂克唑替尼联合治疗显示出临床活性与耐受性^[62]^。

#### 3.2.2 继发*RET*融合

陆续有报告指出，在EGFR抑制治疗过程中，*RTK*融合可以发展成为对EGFR TKI的获得性耐药，而确切发生机制仍有待阐明。对*EGFR*突变NSCLC中作为获得性耐药机制的*RTK*融合类型及其融合伴侣的首个且最全面的病例分析发现，接受1代-3代EGFR TKI治疗后出现获得性耐药RTK融合的86例患者中，*RET*最为常见（43%），*ALK*次之（26%）^[63]^。尽管*KIF5B-RET*为肺癌中检出的*RET*融合变异的最常见形式，在EGFR-TKI治疗进展的病例中仅1例*KIF5B-RET*，占全部获得性耐药*RET*融合的2%；*CCDC6-RET*占比最多（58%），其次为*NCOA4*（26%）^[63]^。随后针对奥希替尼治疗失败的12例*EGFR*突变NSCLC检测同样观察到，随治疗出现的RET融合中最常见的为*CCDC6-RET*（42%）、*NCOA4-RET*（33%），*KIF5B-RET*占17%^[64]^。上述结果提示，这些已发现*RET*融合变异或许在EGFR TKI使用前存在的可能性不大^[63]^。作为获得性耐药机制，*RTK*融合在第1或2代EGFR TKI治疗背景下的发生率（1.8%）低于以奥希替尼（3.7%）为代表的第3代^[63]^。这种差异可能归因于第1代或2代EGFR TKI耐药时常出现第2位点T790M突变，或同时受到测序技术的影响。另外，有研究^[65]^在*ALK*阳性NSCLC肿瘤样本中发现*CCDC6-RET*融合，在二线Brigatinib治疗后产生获得性耐药而检出（为该患的第2次肿瘤活检），而此前在阿来替尼一线治疗后的活检中并未发现。这提示*RET*融合均可能导致*EGFR*或*ALK*阳性NSCLC的获得性耐药。

针对特定获得性耐药*RTK*融合与基础*EGFR*突变的双重阻断已证实了临床安全性和有效性。对于EGFR-TKI耐药后出现*RET*融合的患者，使用卡博替尼或普拉替尼+一种EGFR TKI已证实临床获益^[63]^。已有研究^[66]^表明普拉替尼联合奥希替尼能够克服奥希替尼耐药，迅速且显著改善病情，耐受性良好。塞尔帕替尼+奥希替尼也显示为*EGFR*突变NSCLC出现获得性*RET*融合情况下的可行方案，该联合方案证实为奥希替尼耐药患者带来影像学缓解和持久获益^[64]^。

### 3.3 正在开展的临床试验和新型靶向药物

正在开展的AcceleRET Lung Ⅲ期临床研究旨在比较普拉替尼*vs*标准方案（含铂化疗±帕博利珠单抗）一线治疗晚期或转移性*RET*融合阳性NSCLC的有效性与安全性^[67]^。另一项类似设计的全球随机对照Ⅲ期研究LIBRETTO-431将评估塞尔帕替尼*vs*培美曲塞+顺铂或卡铂±帕博利珠单抗在局部晚期或转移性*RET*融合阳性NSCLC中的一线治疗^[68]^。上述两项研究均允许随机分配至对照组的患者在疾病进展后交叉至RET-TKI组治疗。研究将进一步为RET抑制剂在*RET*融合阳性晚期NSCLC的一线应用提供数据。此外，靶向治疗联合化疗或抗血管生成等治疗模式也值得在未来研究中探索。

RET抑制剂一线治疗*RET*融合阳性NSCLC出现疾病进展后的治疗方案尚需探索，需要进一步观察耐药的机制。Ⅱ期ORCHARD平台研究将入组奥希替尼一线治疗时疾病进展的局部晚期/转移性*EGFR*突变NSCLC，其中组A包括携带*RET*融合的患者并将给予塞尔帕替尼+奥希替尼治疗^[69]^。除了通过联合治疗策略克服耐药，还需要开发下一代RET-TKI以克服门卫和非门卫突变。目前研发中的小分子抑制剂，如①TPX-0046：选择性RET/SRC抑制剂，在RET变异的细胞系和患者来源的异种移植肿瘤模型中已显示出临床前活性^[70]^；②BOS172738：新型RET抑制剂，Ⅰ期研究已经启动^[71]^；③RXDX105：MKI，能够抑制野生型RET、突变型RET（如RET M918T）、由*RET*融合产生的嵌合癌基因蛋白（KIF5B-RET、CCDC6-RET、NCOA4-RET、PRKAR1A-RET），对VEGFR无活性。在Ⅰ期/Ⅰb期研究中，对既往未接受过RET抑制剂的*RET*融合阳性肺癌患者显示出临床活性^[72]^。

## 4 结语

*RET*融合阳性NSCLC的治疗已进入靶向治疗的时代，MKI类药物疗效有限，不良反应发生率高，可作为不能获得RET-TKI治疗后的选择。传统化疗疗效相对有限，免疫治疗单药或联合化疗的疗效欠佳，无论PD-L1表达水平如何，均不推荐使用。

选择性RET-TKI普拉替尼和塞尔帕替尼从根本上改变了*RET*融合阳性NSCLC患者的治疗格局。ARROW研究结果显示，普拉替尼治疗初治和经治*RET*融合阳性晚期NSCLC疗效确切，耐受性良好。普拉替尼在中国已获批用于既往接受过含铂化疗的*RET*融合阳性的局部晚期或转移性NSCLC成人患者的治疗。NCCN指南推荐*RET*融合阳性晚期NSCLC一线首选普拉替尼和塞尔帕替尼治疗。

未来仍需进一步探索*RET*融合阳性晚期NSCLC接受RET抑制剂治疗后的耐药机制，探索同时抑制RET和并行的耐药信号通路的联合治疗新策略，以及积极评估正在研发中的选择性RET-TKI，以期为RET融合阳性NSCLC人群提供更多的治疗选择，为具体患者制定最佳的治疗方案。
